# Numerical study of the influence of water evaporation on radiofrequency ablation

**DOI:** 10.1186/1475-925X-12-127

**Published:** 2013-12-10

**Authors:** Qing Zhu, Yuanyuan Shen, Aili Zhang, Lisa X Xu

**Affiliations:** 1 State Key Laboratory of Oncogenes and Related Genes, School of Biomedical Engineering, Med-X Research Institute, Shanghai Jiao Tong University, Shanghai, China; 2 National-Regional Key Technology Engineering Laboratory for Medical Ultrasound, Department of Biomedical Engineering, School of Medicine, Shenzhen University, Shenzhen 518060, China

**Keywords:** Radiofrequency ablation, Water evaporation, Water diffusion, Mathematical model

## Abstract

**Background:**

Radiofrequency ablation is a promising minimal invasive treatment for tumor. However, water loss due to evaporation has been a major issue blocking further RF energy transmission and correspondently eliminating the therapeutic outcome of the treatment.

**Method:**

A 2D symmetric cylindrical mathematical model coupling the transport of the electrical current, heat, and the evaporation process in the tissue, has been developed to simulate the treatment process and investigate the influence of the excessive evaporation of the water on the treatment.

**Results:**

Our results show that the largest specific absorption rate (*Q*_*SAR*_) occurs at the edge of the circular surface of the electrode. When excessive evaporation takes place, the water dehydration rate in this region is the highest, and after a certain time, the dehydrated tissue blocks the electrical energy transmission in the radial direction. It is found that there is an interval as long as 65 s between the beginning of the evaporation and the increase of the tissue impedance. The model is further used to investigate whether purposely terminating the treatment for a while allowing diffusion of the liquid water into the evaporated region would help. Results show it has no obvious improvement enlarging the treatment volume. Treatment with the cooled-tip electrode is also studied. It is found that the cooling conditions of the inside agent greatly affect the water loss pattern. When the convection coefficient of the cooling agent increases, excessive evaporation will start from near the central axis of the tissue cylinder instead of the edge of the electrode, and the coagulation volume obviously enlarges before a sudden increase of the impedance. It is also found that a higher convection coefficient will extend the treatment time. Though the sudden increase of the tissue impedance could be delayed by a larger convection coefficient; the rate of the impedance increase is also more dramatic compared to the case with smaller convection coefficient.

**Conclusion:**

The mathematical model simulates the water evaporation and diffusion during radiofrequency ablation and may be used for better clinical design of radiofrequency equipment and treatment protocol planning.

## Background

Radiofrequency ablation (RFA) is one of the promising therapeutic procedures of primary and secondary malignant tumor due to its minimal invasiveness, less side-effect and immunology stimulation as compared with other treatment modalities [1,2]. The high frequency alternating electrical current forces agitation of the ions in the tissue, resulting in frictional heat of the tissue and correspondent abrupt increase of temperature. Previous studies show that, when the local temperature reaches the boiling temperature of water 100°C, excessive evaporation takes place and increases the tissue impedance [3]. The roll-off has been a major obstacle for its application [4], especially for tumors larger than 3 cm [5-8]. Studies have been focused on improving therapeutic effect of RFA, either through better design of the device (for example, shape/geometry of electrodes, cooled-tip electrode) [9,10], or optimizing the RF treatment protocols [11,12].

Multiple probes, wet electrode and cooled-tip electrode are techniques proposed to enlarge the lesion size [13-16]. These designs have been proven to be able to improve the therapeutic outcome, however a better design and planning of the treatment protocols is still needed for clinical applications of RF. When multiple probes create enlarged coagulation volume, there may exist survived tumor cells in the intermediate region of the multiple lesions [15,16]. Design of the wet electrode enables perfusion of the hypertonic saline solution into the tumor region with uncontrollable and irregular enlarged treatment area [17,18]. Design of the cooled-tip electrodes allows circulation of the cooling agents inside the electrode, which helps lower the temperature on the contact surface, thus slows down local water loss rate, and eventually increases the coagulation volume up to 50% [19]. However there also exists regions with evaporation which blocks the RF energy transportation [20,21]. The existence of the dehydration of the tissue not only leads to the final formation of charring and decreases the electrical conductivity of the tissue, it also adsorbs large quantity of energy. Understanding how the evaporation process influences the transportation of the electrical currents [9] and thus adsorption of the energy will be the key issue to be able to further optimize the treatment protocols.

Mathematical modeling has been an efficient and low cost modality in both design and treatment planning [22]. Models have been developed to simulate the temperature distributions of tissue during the treatment [4,23-26]. The complexity of the tumor composition, the influence of heterogeneous electrical conductivity, thermal conductivity, blood perfusion rate, and the specific distribution of blood vessels on the results have been studied [27-35].

However, most of the models have skipped the period when the excessive evaporation taking place during their simulation. Ai et al. [36] have incorporated the latent heat of evaporation into the specific heat capacity of the tissue in the bioheat transfer equation but diffusion of the water has been neglected. Yang et al. have proposed an empirical equation considering the influence of the evaporation process [37]. The dependence of the liver water content has been measured and a function describing this dependence established based on the experimental findings. The evaporation heat is further determined from the decreasing rate of liquid water fraction, and added as the heat sink in the heat transfer equation [38]. Both of these two models have ignored the influence of water loss on the electrical field and heat transfer as well. Patz et al. [39,40] has modeled the process of gas bubbles emerging, transportation, and merging with each other during the RF treatment. In their model, as soon as the tissue reached the boiling temperature, evaporation takes place, and an interface is immediately formed separating the gas region and the liquid region, which is inconsistent with Haemmerich’s experiment findings [38].

In this study, a numerical model is developed investigating transportation of the electrical currents and heat inside the tissue during RF heating. Local boiling phenomena (evaporation) and diffusion of the water are both considered. These processes are coupled together. It is expected to help accurately predict deposition of RF energy before or after carbonization, and thus for effective prevention as needed. Also numerically studied is simulation of the treatment using different treatment protocols with the cooled-tip electrode.

### Theoretical modeling

**Figure 1:**
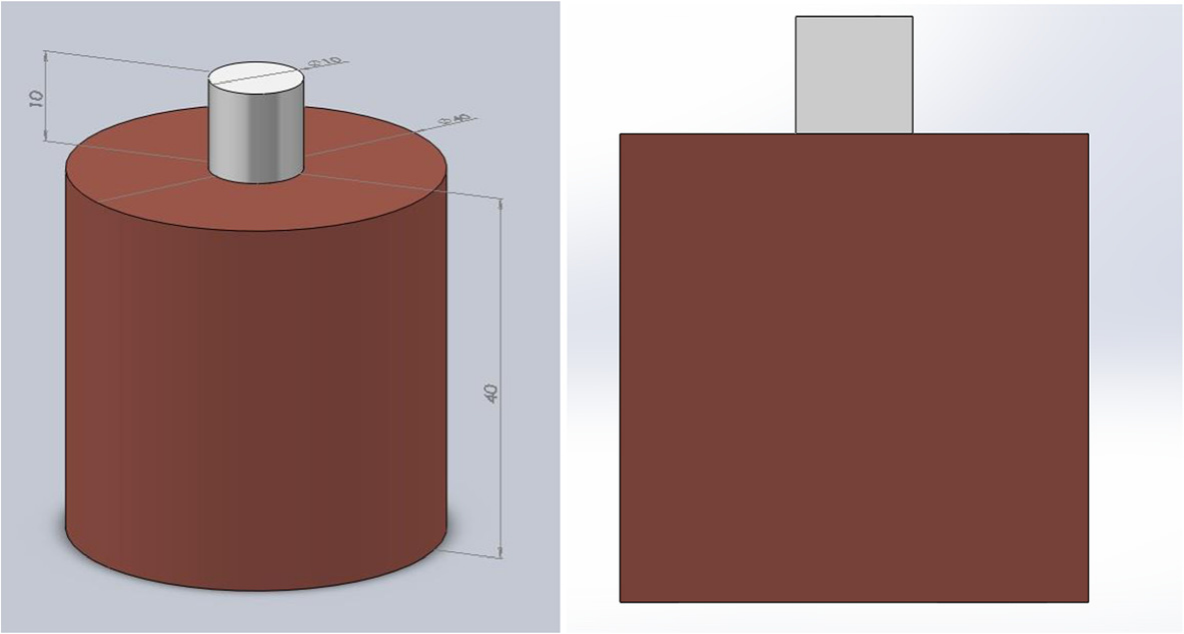
**Schematic diagram of the eletrode and tissue.** The gray cylinder represents the RF electrode, while the dark red cylinder represents the tissue.

The electrodes of RFA used in this model are similar to those as described previously [41]. The geometry of the tissue and the electrode are illustrated in Figure 1. The treating surface of the electrode is a 10 mm diameter circle. The grounding pad is placed at the bottom of tissue. The tissue is assumed to be a symmetric cylinder with a diameter of 40 mm and a height of 40 mm, which is estimated from a typical Wistar mouse’s height and the width. And the tissue is assumed to be homogenous. Treating face of the electrode closely contacts the upper surface of tissue. The tissue and the electrode are assumed to be axially symmetric cylinders, thus only changes in the axial and radial directions are considered. So it is simplified to a 2-D model, and cross-section of electrode and tissue is studied.

The RF frequency used is 460 kHz. As the wavelength of the electromagnetic field is much longer than the electrode, the quasi-static approximation of the electrical field in the tissue is applied [26,42],

(1) $$\nabla \sigma_{t}\nabla V=0$$

where *V* is the electrical potential, and *σ*_t_ is the electrical conductivity of tissue.

Neumann boundary condition is used to describe the insulated boundary of tissue and the electrode:

∇V=0

The absorption rate of RF energy in tissue, *Q*_*SAR*_ (W/m^3^) is,

(2) $$Q_{\mathrm{SAR}}=\sigma_{t}{\left(\nabla V\right)}^{2}$$

Pennes’ equation is used to describe the heat transfer inside the tissue [43],

(3) $$\rho_{t}c_{t}\frac{\partial T_{t}}{\partial t}=\nabla k_{t}\nabla T_{t}+\rho_{b}c_{b}w_{b}\left(T_{b}-T_{t}\right)+Q_{\mathrm{meta}}+Q_{\mathrm{SAR}}$$

where *ρ*_*t*_, *c*_*t*_ , *k*_*t*_ stand for the density, heat capacity and thermal conductivity of the tissue; *T*_*b*_, *w*_*b*_ , *ρ*_*b*_ are the arterial blood temperature, blood perfusion rate, the density of blood; *Q*_*meta*_ is the metabolic rate of tissue, and *Q*_*SAR*_ is the specific absorption rate of tissue.

A natural convection boundary condition is used for the outer surface of the tissue cylinder which includes the upper surface excluding the contact surface between the electrode and tissue, the side surface, and the bottom surface of the modeled tissue cylinder, as shown in Figure 1,

(4) $$k\nabla T{|}_{\mathrm{boundary}}=h_{s}\left(T_{\mathrm{air}}-T_{t}\right)$$

where *h*_*s*_ is the natural convection coefficient of air, and a value of 25 W/m^2^K is used.

The blood flow stops when the local temperature is higher than 50°C [44],

(5) $$w_{b}=\left\{\begin{array}{l} w_{b0}\;T<50^{o}C\\ 0\;T\geq 50^{o}C \end{array}\right)$$

And the metabolic rate of tissue is lineally dependent on temperature [45],

(6) $$Q_{\mathrm{meta}}=\left\{\begin{array}{l} Q_{\mathrm{meta}0}\left[1+10\%\left(T-T_{0}\right)\right]\;T\leq 50^{o}C\\ 0\;T>50^{o}C \end{array}\right)$$

where *Q*_*meta0*_ is the metabolic rate in physiological state (T_0_ = 37°C).

With temperature rising, ions and polar molecules in the tissue oscillate faster, which helps heat transfer and electromagnet field propagate. The physical phenomenon reflects in the changes of the thermal and electrical conductivity [46]. Their dependence on temperature has been studied by Bhattacharya [47] and Pop [48] experimentally. And it can be described by a linear function with an independent variable—temperature,

$$k\left(T\right)=k_{0}*\left[1+0.3\%\left(T_{t}-T_{0}\right)\right]*10^{-1}$$

(7) $$\sigma \left(T\right)=\sigma_{0}\left[1+0.016\left(T-T_{0}\right)\right]\;$$

where *T*_0_ is the reference temperature which is the initial temperature of tissue, usually the body temperature; *σ*_*0*_ and *k*_*0*_ are the electrical conductivity and thermal conductivity at *T*_*0*_, separately.

As heating continues, temperature of the surface contacting with the electrode will reach the boiling point of water. According to Webster’s experimental study [37], the excessive evaporation inside the tissue takes place between 100°C and 105°C. Therefore, the boiling temperature of pure water under 1 atm, 100°C, is used as the criterion for the occurrence of the evaporation inside the tissue. Thus according to energy conservation,

(8) $$Q_{\mathrm{eva}}=-\left(Q_{\mathrm{SAR}}+\nabla k_{t}\nabla T_{t}\right)$$

where *Q*_*eva*_ is the energy absorbed by evaporation per unit volume of tissue.

And the dynamic volume fraction of liquid water in tissue is,

(9) $$\frac{dm_{l}}{\mathrm{dt}}=\frac{Q_{\mathrm{eva}}}{h_{\mathrm{fg}}}+\nabla D_{l}\nabla m_{l}$$

where *m*_*l*_ is the mass of liquid water per unit volume of tissue, h_*fg*_ is the latent heat of water, *Q*_*eva*_ is the heat absorbed per unit volume of tissue by evaporation, and *D*_*l*_ is the water diffusion coefficient, which exponentially increases with temperature according to Mill’s [49] and Krynicki’s study [49,50],

(10) $$D_{l}=12.5*10^{-9}\exp\left(-5.22*10^{-4}\right)\exp\left(\frac{-925\exp\left(-2.6*10^{-4}\right)}{T_{t}-\left(95+2.61*10^{-2}\right)}\right){T_{t}}^{\frac{1}{2}}\;m^{2}/s$$

The liquid water is considered as incompressible and the tissue volume assumed to be constant during the treatment. After evaporation, the space previously taken by the liquid water in tissue is filled with steam. The volume is occupied by a liquid–gas mixture. The liquid and gas fractions of water could be determined by:

(11) $$\phi_{w}=\frac{m_{l}}{m_{l0}}$$

(12) $$\phi_{g}=1-\phi_{w}=1-\frac{m_{l}}{m_{l0}}$$

where *ϕ*_*g*_ and *ϕ*_*w*_ are the fractions of water in gas and liquid phase, *m*_*l0*_ is the initial mass of liquid water per unit volume of tissue.

The modified equation describing the electrical conductivity of the two-phase (liquid and gas) system is used [51]:

$$\frac{\sigma_{t}\left(T,m_{l}\right)}{\sigma \left(T\right)}=\frac{1+\mathrm{AB}\phi_{g}}{1-\mathrm{B\psi}\phi_{g}}$$

(13) $$\psi =1+\left(\frac{1-\phi_{m}}{\phi_{m}^{2}}\right)\phi_{g}$$

where *A* is a constant that primarily depends on the shape and the orientation of dispersed particles, for randomly distributed bubbles in tissue, A is 1.5; *B* is a constant which represents the relative conductivity of the two components, and the value is 2/3 for water and water vapor; *ϕ*_*m*_ is the maximum packing fraction of particles, and it is 0.637 for randomly packed sphere gas bubbles.

Thermal conductivity of the mixture is a linear function of water concentration [52]:

(14) $$k_{t}\left(m_{l},T\right)=k\left(T\right)*\left(0.133+1.36\frac{m_{l}}{m_{l0}}\right)$$

The electrical and thermal conductivities of the tissue depend both on temperature and the water content, the influence of both factors are assumed to be independent of each other, thus Eq. (15) is proposed to describe its dependence on both temperature and water content:

$$\sigma_{t}\left(T,m_{l}\right)=\sigma_{0}\left[1+0.016\left(T-T_{0}\right)\right]\;\frac{1+\mathrm{AB}\phi_{g}}{1-\mathrm{B\psi}\phi_{g}}$$

(15) $$k_{t}\left(m_{l},T\right)=4.19*\;\left[1+0.3\%\left(T_{t}-T_{0}\right)\right]*10^{-1}*\left(0.133+1.36\frac{m_{l}}{m_{l0}}\right)$$

The equations describing the electrical field, heat transfer and water evaporation Eq. (1)-Eq. (10) are solved by Comsol 4.2. The equations of thermal and electrical conductivities Eq. (11)-Eq. (15) are solved by Matlab7.0b, with the results input to Comsol to couple with the electrical and thermal equations at the same time. The choice of time step is determined by taking both computational convergence and efficiency into consideration. And 0.1 s and 0.01 s are chosen for the simulation process before and after the occurrence of evaporation respectively. Before the beginning of the evaporation (boiling), only Eq. (1) –Eq. (7) are needed to be solved. A time step of 0.1 s is small enough for convergence. When the evaporation takes place, Eq. (8)-Eq. (15) are evolved, as excessive changes taking place in a short time, thus after several trials, a time step of 0.001 s is chosen for this period.

## Results and discussion

**Figure 2:**
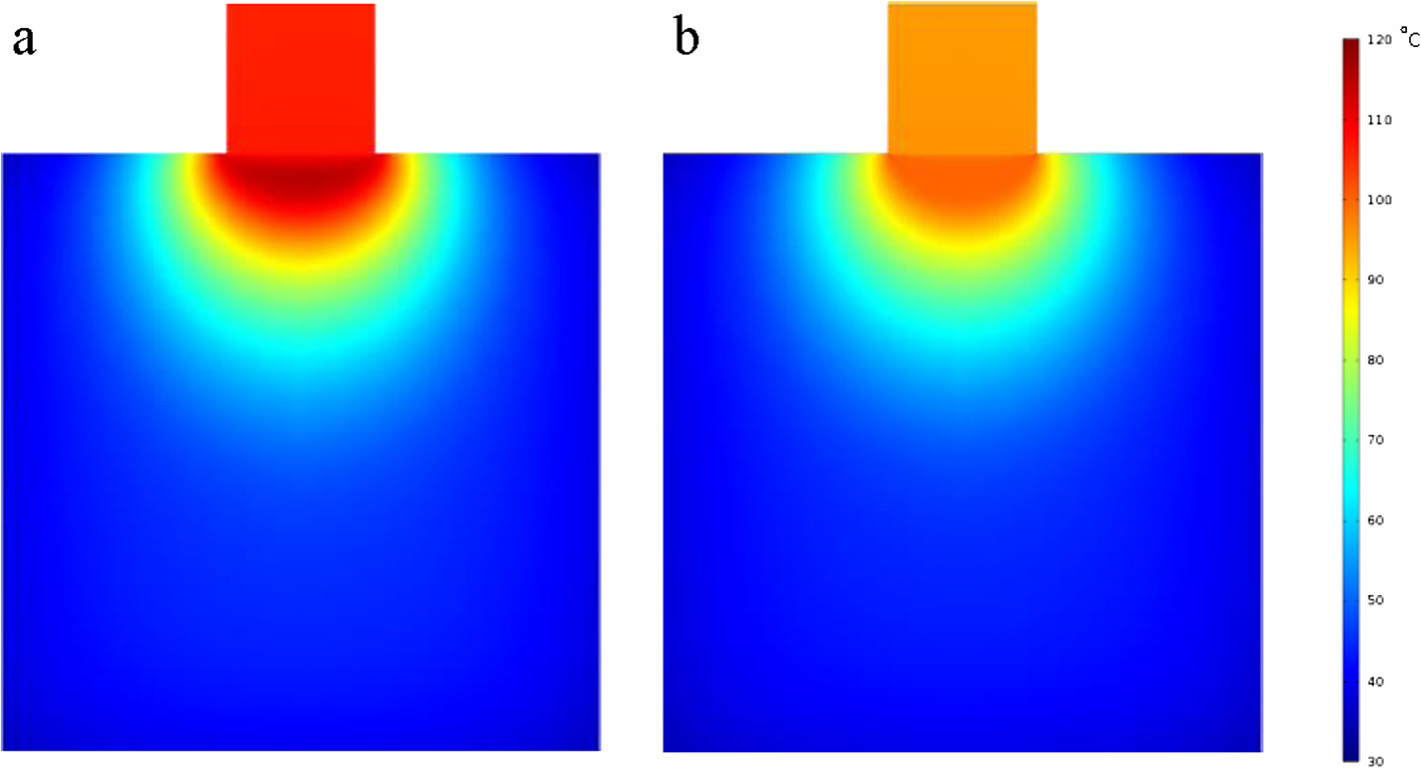
**Temperature distribution of two models at t = 600 s. (a)** Temperature distribution without considering evaporation in the model at t = 600 s; **(b)** Temperature distribution with considering evaporation in the model at t = 600 s.

**Figure 3:**
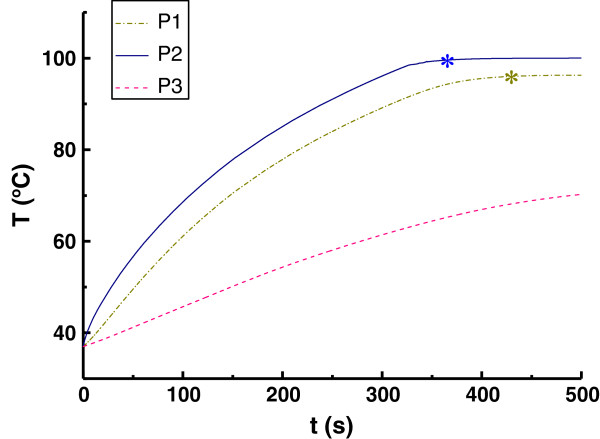
**Transient temperatures at the monitoring points.** P1, P2, P3 locate at the central axis of the tissue cylinder with 0 mm, 1 mm and 10 mm away from the contact surface of the electrode.

**Figure 4:**
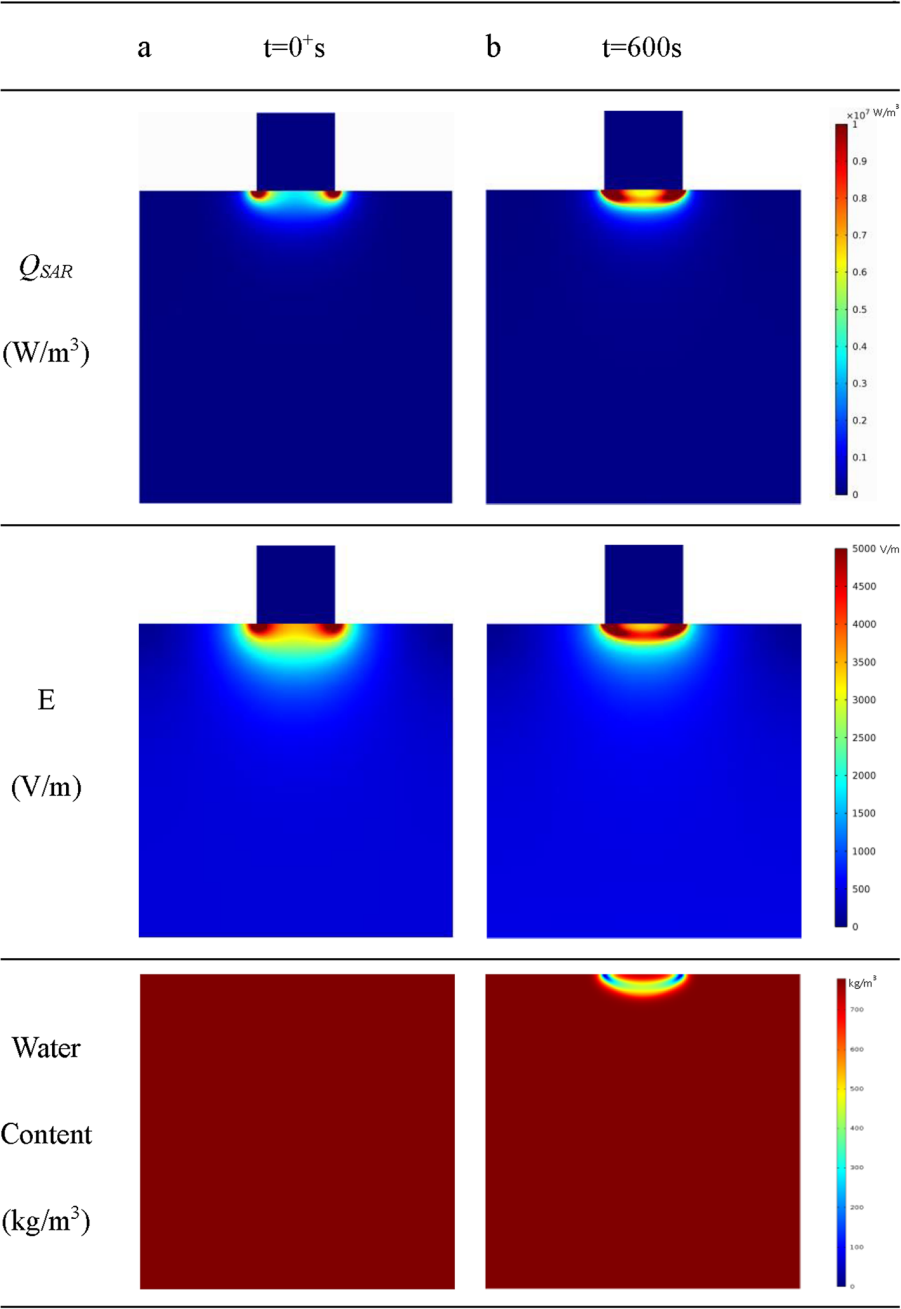
***Q***_***SAR***_**, electrical field and water content distributions of tissue. (a)** The *Q*_*SAR*_, electrical field and water content distribution at the beginning; **(b)** The pattern of *Q*_*SAR*_, electrical field and water content distribution after heating for 600 s with considering evaporation.

**Figure 5:**
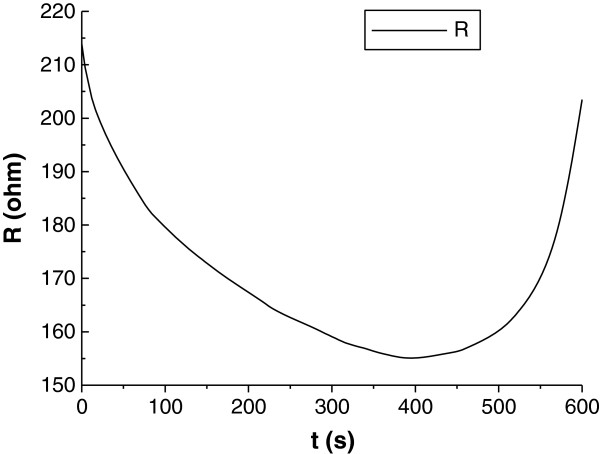
**The tissue impedance during treatment.** X-axis stand for the time of RF treatment, Y-axis stands for the tissue impedance.

The parameters used in the model are listed in Table 1. The simulated transient temperature profiles, water content and the electrical field intensity inside tissue are shown in Figures 2, 3, 4 and 5. For comparison, the results obtained without considering the evaporation process are present in Figure 2a. They are obtained by deleting the water evaporation part in Eq. (8)-Eq. (14).

**Table 1:** Parameters used in the model

**Parameter**	**Symbol**	**Value**	**Ref.**
Blood temperature	T_b_	37°C	
Atmosphere temperature	T_air_	20°C	
Metabolic rate	Q_meta0_	33800 W/m^3^	[53]
Latent of phase change	h_fg_	2.26E6 J/kg	[53]
Constant of mixture	A	1.5	[51]
Constant of mixture	B	-2/3	[51]
Constant of mixture	*ϕ*_*m*_	0.637	[51]
Electrical conductivity	*σ*_*0*_	0.336 S/m	[42,54]
Blood perfusion (volume rate of blood flow per unit volume of tissue)	*w*_*b*_	0.008 s^-1^	[42]
Original water fraction in the tissue (mass of water per unit volume of tissue)	*m*_*l0*_	778 kg/m^3^	[38]
Electrode voltage	*V*	35 V	

The temperature distribution at t = 600 s is illustrated in Figure 2. It is found that the simulated highest temperature reaches 119.79°C if evaporation is not considered, while the highest temperature inside tissue is about 100.24°C when the evaporation process considered. If using 50°C as the critical temperature assessing cell viability, it can be found from the results that without considering the evaporation process, the 2-D lesion area will be 12.41% larger. Obviously, the model without considering evaporation overestimates the temperature profile in tissue and correspondently the tumor lesion size. The transient temperature curves at the monitoring points are illustrated in Figure 3. The points are at the center line of the tissue cylinder with 0 mm, 1 mm, and 10 mm away from the contact surface. As seen from Figure 3, the temperatures at the first and the second monitor points which are closer to the contact surface (0 mm, 1 mm) keep rising before the temperature reaches a turning point, where the temperature starts to keep at a constant value 100°C, with the adsorbed RF energy being exhausted by evaporation. The temperature at the third point which is 10 mm away from the contact surface keeps rising during the whole RF heating process, and no evaporation takes place at this point.

To find out how the evaporation process influences the transportation of RF energy, the electrical field *E* and *Q*_*SAR*_ of tissue are obtained, and results are illustrated in Figure 4. The field of the specific heat adsorption rate *Q*_*SAR*_ and the electrical field intensity *E* are established at the instant the RF treatment begins (Figure 4a). The largest electrical field intensity locates at the edge of the electrode contact surface, and the *E*-lines form semi-circle shapes with their centers at the edge of the electrode. After being heated for 600 s, though the locations with the largest value of *Q*_*SAR*_ and the electrical field intensity are still at the edge of the electrode, the shapes of the *E*-lines are no longer semi circles (Figure 4b). Comparing with the distributions at the time point *t = 0+*, the electric field in the tissue after being heated for 10 minutes is more concentrated in the contacted area. And as shown in Figure 3, at this time point, excessive evaporation has taken place, it is the evaporation process that has reshaped the distribution of the *Q*_*SAR*_ and electrical intensity *E*.

Also shown in Figure 4, the region with the largest *Q*_*SAR*_ value is the place where the liquid water loses the most. With decrease of the water content in this region, the local electrical conductivity decreases, the water loss region forms a barrier for transportation of the electric currents in the radial direction. The trapped electrical field propagates more in the axial direction near the center line of the cylinder. This explains the unique shape of the *E* lines shown in Figure 4b.

Tissue impedance is one of the most frequently used clinical criteria to decide when to adjust RF power or to stop the treatment [55]. The tissue impedance during the treatment is also calculated with results shown in Figure 5. It slowly decreases at the beginning of the treatment, and after reaches the lowest point (the turning point), it increases quickly. According to the simulated water content results, the water evaporation emerges at about 334 s after the treatment starts, while the reflection point of the impedance increasing from its former decreasing trend starts at 399 s. There is a time interval of about 65 s between the occurrence of water evaporation and the inflection point of the impedance. This may due to the overwhelming influence of temperature on the tissue’s electrical conductivity when the water content is not significantly decreased at the beginning of the heating.

**Figure 6:**
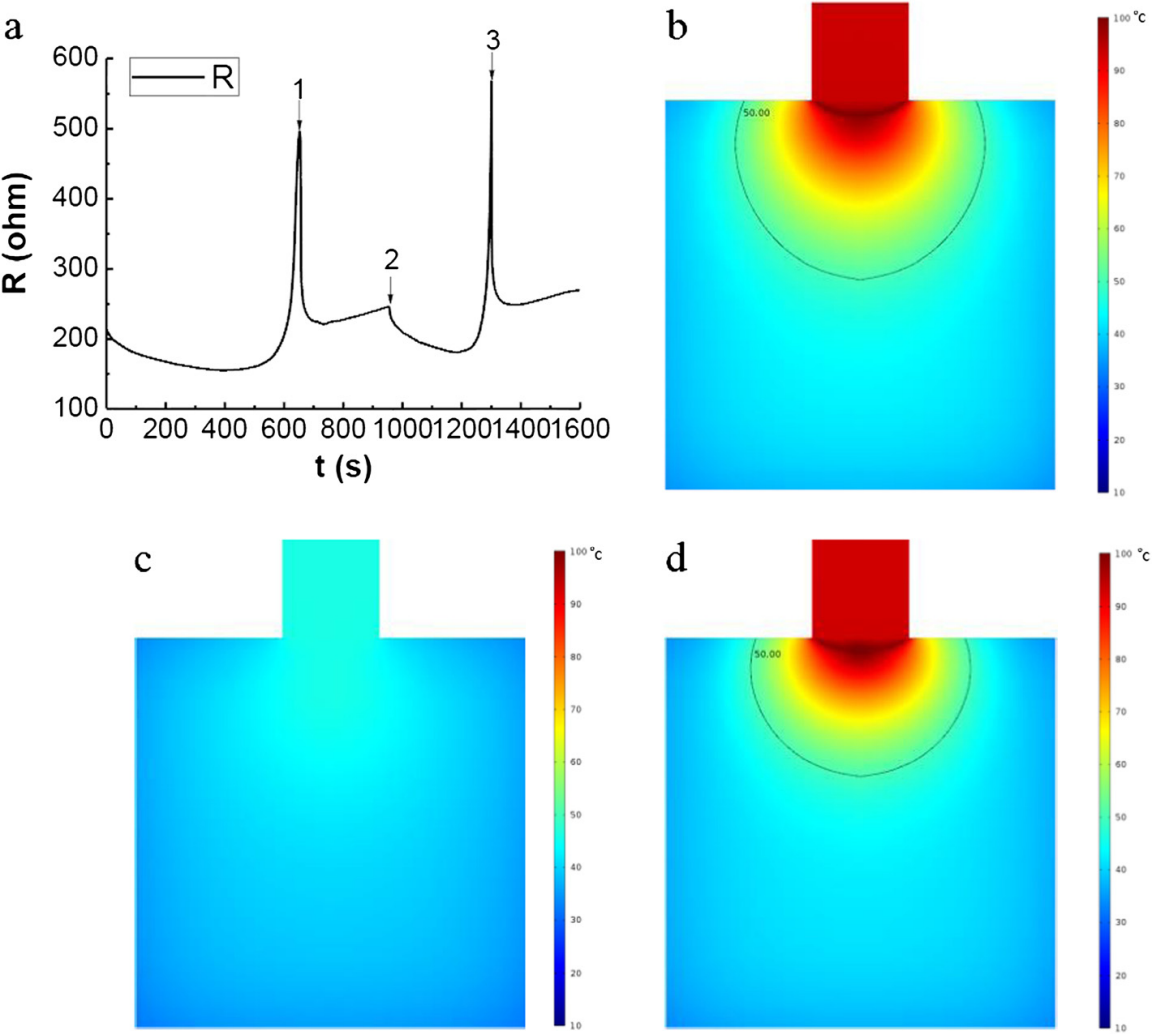
**The tissue impedance via time, and temperature distribution at t = 656 s, 956 s and 1300 s. (a)** Tissue impedance via time of both models. Point 1 is when the first heating stops; point 2 represents the start of the second treatment of RF; point 3 is when the second heating stops; **(b)** Temperature distribution of tissue at the end of first treatment, t = 656 s; **(c)** Temperature distribution of tissue at the beginning of second RF treatment, t = 956 s; **(d)** Temperature distribution at the end of second RF treatment, t = 1300 s.

According to the above simulation results, without adjusting the treatment protocol, there will form regions with serious water loss as barriers for RF energy delivery. However there is an interval between the occurrence of the evaporation and the beginning of the impedance increase. If at a certain time after the evaporation starts, terminating the heating allowing some time for the liquid water in the adjacent region to rehydrate the region may help enlarge the coagulation depth of the treatment. To find out its effectiveness, a heating process as illustrated in Figure 6a is studied. The first RF heating ends at 656 s as the impedance exceeds 500 ohm. Then a 5 min of natural cooling of tissue is allowed before restarting the RF heating. The second heating lasts for 344 s until the impedance exceeds 500 ohm again. During the natural cooling process, it can be seen that the tissue impedance first drops dramatically, and then increases slowly. The change of the impedance is clearly related to the water content inside the tissue. The temperature distribution at these time points (t = 656 s, t = 956 s, t = 1300 s) are given in Figure 6b-d. Unexpected, after the second heating (t = 1300 s), the heated region whose temperature is greater than 50°C is 28.5% less than that resulted from the first heating. This result suggests that by just waiting for the water to flow back to the dehydrated region is not an effective way improving the treatment outcome. During the intermittent thawing process when water flows back (about 64.3% rehydrated after 5 min, results not shown), the local temperature also drops (Figure 6d). The second heating only increases the temperature of the same region to the previous point and fails to further propagate before the gas gap forms again and significantly increases the impedance. Continuously hydrating the water loss region of the tissue without decreasing the local temperature shall be more effective, adding hot sterile saline is an option if the process is well controlled.

To avoid local carbonization due to dehydration, electrodes with cooling agent flowing inside have been used [56,57]. Circulation of the cooling agent dissipates heat from the electrode wall and thus decreases the temperature in the adjacent region, which helps reduce local water evaporation. It has been proved effective in improving thermal ablation outcome of large tumor. To help optimize the treatment protocols, outcome of three different cooling strategies are predicted using the proposed model. The convective coefficient between the electrode wall and the circulating fluid *h*_e_ is assumed to be 25 W/m^2^K, 100 W/m^2^ and infinity. The temperature of the cooling agent is 20°C.

**Figure 7:**
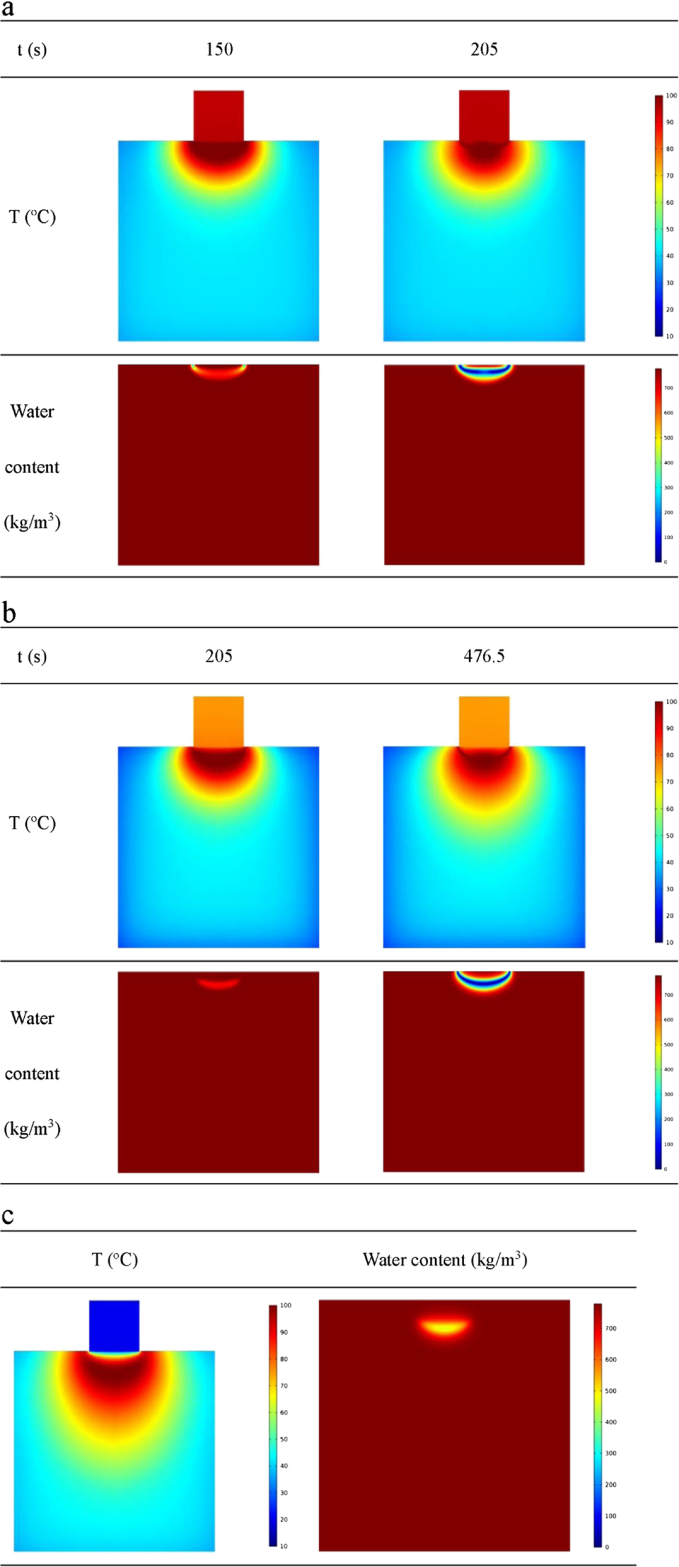
**Temperature and water content distribution at different cooling conditions. (a)** Temperature and water content distributions under low convection coefficient (h = 25 W/m^2^K) at 150 s and 205 s. **(b)** Temperature and water content distributions under medium convection coefficient at 205 s and 476.5 s h = 100 W/m^2^. **(c)** Temperature and water content distribution under constant temperature (T = 20°C) at 1400 s.

The temperature distribution and water content in the tissue during the treatment with these conditions are calculated and illustrated in Figure 7. It is found that during the treatment with the cooled-tip electrodes, there are certain locations inside the tissue whose temperature would reach the boiling point for all the cooling conditions studied, and under the first two conditions (with limited convection coefficient, there would form a complete enclosed gas gap in the tissue (shown in Figure 7a and b). This completely blocks further energy transmission of RF. It takes 205 s and 476.5 s for the complete gas gap to form for the first two cooling conditions, respectively. While for the infinite convective coefficient, that is, the temperature of the electrode wall is maintained at a constant value, thermal equilibrium achieves at 1400 s. Circulation of the cooling agents inside the electrode not only extends the therapy time but also the therapy area. The fraction of the tissue area with temperature higher than 50°C divided by the total tissue area in the 2D-section are 22.0%, 41.5% and 33.9% respectively under the three conditions. It is the largest for the process with moderate convection coefficient (100 W/m^2^K) of the cooling agent.

Another finding is that the flowing condition of the cooling fluids also alters the pattern of water loss. For the flow with a lower convection coefficient, the water loss first occurs at the edge of contact surface. While for the medium and extremely high convection coefficients, the water loss starts from the center of tissue at about 2.5 mm below the contact surface. The results suggest that the treatment region can be shaped by controlling the flowing conditions inside the electrode.

**Figure 8:**
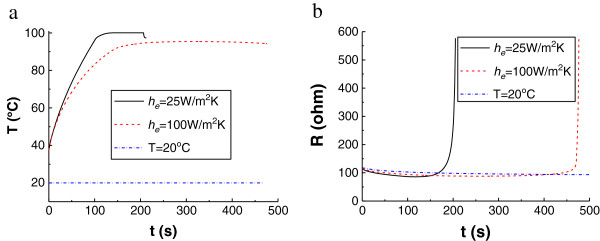
**Transient temperature and impedance via time. (a)** Transient temperature and **(b)** Transient impedance changes at the monitoring point under different treatment conditions.

Further investigation of the transient temperature distribution during the treatment (Figure 8) has found that for some time after evaporation takes place, there is a decrease of the local temperature. The monitoring point in Figure 8a is the center of the contact surface. At the time when the temperature at this point starts to decrease, the gas vapor may have formed a barrier for the electrical field and also decreases the thermal conduction, while at the same time, the cooling agent continuously circulates, and it lowers the local temperature of tissue.

From Figure 8b, it can be found that impedance decreases slightly before the vapor point, and increases suddenly while gas forms a gap. Comparing Figure 8a and b, the turning point achieves at 125 s under the low convection coefficient and 195 s under the medium convection coefficient; and the impedance exceeds 500 ohm at 204 s under the low convection coefficient and 476 s under the medium convection coefficient. The larger the convection coefficient is, the longer the treatment time required.

One interesting phenomenon observed is that, at the end of treatment, the impedance during the treatment with the medium convection coefficient increases more rapidly than that under a lower convection coefficient. As seen from Figure 8b, it costs 10.6 s for the impedance increasing from 115 ohm to 500 ohm under a larger convective coefficient, while it costs 25.6 s under the other condition. The result suggests that when cooled-tip electrode is applied, monitoring the rising of the impedance is necessary.

Besides, though the larger the convection coefficient, the longer the treatment time is, with further increase of the convection coefficient, a lot more heat is dissipated by the convection. When the cooling process exceeds the heating effect of the RF, the ablated size would decrease. There would be an optimal flow conditions inside the probe for a certain shaped ablation requirement. The model presented in this paper is expected to be useful in the treatment planning.

In devices developed in [41,58,59], low temperature nitrogen is used as cooling agent inside the treatment probe. As the nitrogen has a small thermal capacity, the temperature of the flow also changes, by coupling with the flow and heat transfer of the fluid, the model could be used for its better planning.

However, in the model, free diffusion of water is used, and the effect of the tissue structure on the diffusion would result in slower liquid water diffusion, and correspondently an earlier forming of a complete gas region. The pressure difference in tissue is also neglected. With further understanding of gas and water transport during the evaporation, the model will be more accurate to predict temperature above 100°C and dynamical impedance variation. With the patient information of tumor geometry and vessel distribution by imaging technique, it is possible to predict temperate distribution by RFA, and to optimize the placement of electrode.

## Conclusions

In this study, a model incorporating evaporation, water diffusion and its influence on thermal and electrical conductivity has been developed to simulate the RF treatment. The distribution of the temperature, electrical field, *Q*_*SAR*_ and water content in the tissue under different treatment conditions have been obtained numerically. The results show that the evaporation will not only maintain the highest temperature of tissue at 100°C, but also influence the distribution of the electrical field and the *Q*_*SAR*_. A time lag between the emerging of the evaporation and increase of the tissue impedance is found, which is consistent with others’ experiment findings.

It is also predicted that terminating the heating for a while to allow time for liquid water to flow back to the dehydrated region during the treatment would not enlarge the coagulation zone. The numerical results also confirm the advantage of the cooled-tip electrode. Increase in the convective coefficient of the circulating cooling agent inside the electrode would extend the treatment time, reduce the occurrence of the local evaporation and reshape the treated region, but not necessarily enlarge the size of the ablated region. Water loss emerges at different region of tissue under different convection condition. And the tissue impedance would increase more sharply before charring occurs under the medium convection condition. Thus, it is necessary to monitor the increase rate of impedance during the treatment.

## Abbreviations

RFA: Radiofrequency ablation; RF: Radiofrequency; SAR: Specific absorption rate.

## Competing interests

The authors declare that they have no competing interests.

## Authors’ contributions

QZ developed the mathematical model, carried out numerical simulations, and drafted the manuscript. YS participated in code debug and manuscript revision; AZ and LX instructed model development, results interpretation and manuscript writing and revision. All authors read and approved the final manuscript.

## References

[B1] Ahmed M Goldberg S Mueller P, Adam A Principles of radiofrequency ablation Interventional Oncology 2012 New York: Springer 23 37

[B2] Calderwood SK Keisari Y Hyperthermia, the tumor microenvironment and immunity Tumor Ablation. Volume 5 2013 Netherlands: Springer 29 37 *The Tumor Microenvironment*

[B3] Haines DE Verow AF Observations on electrode-tissue interface temperature and effect on electrical impedance during radiofrequency ablation of ventricular myocardium Circulation 1990 12 1034 1038 10.1161/01.CIR.82.3.1034 2393987

[B4] Trujillo M Alba J Berjano E Relationship between roll-off occurrence and spatial distribution of dehydrated tissue during RF ablation with cooled electrodes Int J Hyperther 2012 12 62 68 10.3109/02656736.2011.631076 22235786

[B5] Nagtegaal ID Quirke P What is the role for the circumferential margin in the modern treatment of rectal cancer? J Clin Oncol 2008 12 303 312 10.1200/JCO.2007.12.7027 18182672

[B6] Hines-Peralta A Hollander CY Solazzo S Horkan C Liu ZJ Goldberg SN Hybrid radiofrequency and cryoablation device: preliminary results in an animal model J Vasc Interv Radiol 2004 12 1111 1120 10.1097/01.RVI.0000136031.91939.EC 15466798

[B7] Ring ME Huang SK Gorman G Graham AR Determinants of impedance rise during catheter ablation of bovine myocardium with radiofrequency energy Pacing Clin Electrophysiol 1989 12 1502 1513 10.1111/j.1540-8159.1989.tb06155.x 2476779

[B8] Stein T Untersuchungen zur Dosimetrie der hochfrequenzstrominduzierten interstitiellen thermotherapie in bipolarer technik Ecomed Press 2000

[B9] Ni Y Mulier S Miao Y Michel L Marchal G A review of the general aspects of radiofrequency ablation Abdom imagingI 2005 12 381 400 10.1007/s00261-004-0253-9 15776302

[B10] Zhang XT Zhu SA He B Imaging electric properties of biological tissues by RF field mapping in MRI IEEE T Med Imaging 2010 12 474 481 10.1109/TMI.2009.2036843 PMC2841327 20129847

[B11] Ahmed M Liu Z Humphries S Goldberg SN Computer modeling of the combined effects of perfusion, electrical conductivity, and thermal conductivity on tissue heating patterns in radiofrequency tumor ablation Int J Hyperther 2008 12 577 588 10.1080/02656730802192661 18608580

[B12] Haemmerich D Wright AW Mahvi DM Lee FT Jr Webster JG Hepatic bipolar radiofrequency ablation creates coagulation zones close to blood vessels: a finite element study Med Biol Eng Comput 2003 12 317 323 10.1007/BF02348437 12803297

[B13] Crocetti L de Baere T Lencioni R Quality improvement guidelines for radiofrequency ablation of liver tumours Cardiovasc Inter Rad 2010 12 11 17 10.1007/s00270-009-9736-y PMC2816824 19924474

[B14] Solbiati L Goldberg SN Ierace T Livraghi T Meloni F Dellanoce M Sironi S Gazelle GS Hepatic metastases: percutaneous radio-frequency ablation with cooled-tip electrodes Radiology 1997 12 367 373 9356616 10.1148/radiology.205.2.9356616

[B15] Meijerink MR van den Tol P van Tilborg AAJM van Waesberghe JHTM Meijer S van Kuijk C Radiofrequency ablation of large size liver tumours using novel plan-parallel expandable bipolar electrodes: initial clinical experience Eur J Radiol 2011 12 167 171 10.1016/j.ejrad.2009.06.025 19616911

[B16] Lee J Lee JM Yoon JH Lee JY Kim SH Lee JE Han JK Choi BI Percutaneous radiofrequency ablation with multiple electrodes for medium-sized hepatocellular carcinomas Korean J Radiol 2012 12 34 43 10.3348/kjr.2012.13.1.34 22247634 PMC3253401

[B17] Kettenbach J Kostler W Rucklinger E Gustorff B Hupfl M Wolf F Peer K Weigner M Lammer J Muller W Goldberg SN Percutaneous saline-enhanced radiofrequency ablation of unresectable hepatic tumors: initial experience in 26 patients AJR Am J Roentgenol 2003 12 1537 1545 10.2214/ajr.180.6.1801537 12760914

[B18] Kim JH Kim PN Won HJ Shin YM Percutaneous radiofrequency ablation using internally cooled Wet electrodes for the treatment of hepatocellular carcinoma Am J Roentgenol 2012 12 471 476 10.2214/AJR.11.6583 22268196

[B19] Watanabe I Masaki R Min N Oshikawa N Okubo K Sugimura H Kojima T Saito S Ozawa Y Kanmatsuse K Cooled-tip ablation results in increased radiofrequency power delivery and lesion size in the canine heart: importance of catheter-tip temperature monitoring for prevention of popping and impedance rise J Interv Card Electr 2002 12 9 16 10.1023/A:1014140104777 11839878

[B20] Hong K Georgiades C Radiofrequency ablation: mechanism of action and devices J Vasc Interv Radiol 2010 12 S179 S186 10.1016/j.jvir.2010.04.008 20656227

[B21] Mah YH Ng KH Abdullah BJJ Kwek KH Wong JHD Dossel O, Schlegel WC Ex vivo experiment of bovine liver using cool-tip (TM) radiofrequency ablation system World Congress on Medical Physics and Biomedical Engineering, Vol 25, Pt 6. Volume 25 2009 New York: Springer 209 212 *IFMBE Proceedings*]

[B22] Berjano EJ Theoretical modeling for radiofrequency ablation: state-of-the-art and challenges for the future Biomed Eng Online 2006 12 24 40 10.1186/1475-925X-5-24 16620380 PMC1459161

[B23] Haemmerich D Tungjitkusolmun S Staelin ST Lee FT Mahvi DM Webster JG Finite-element analysis of hepatic multiple probe radio-frequency ablation IEEE T Bio-Med Eng 2002 12 836 842 10.1109/TBME.2002.800790 12148822

[B24] Laeseke PF Sampson LA Haemmerich D Brace CL Fine JP Frey TM Winter TC Lee FT Multiple-electrode radiofrequency ablation: simultaneous production of separate zones of coagulation in an in vivo porcine liver model J Vasc Interv Radiol 2005 12 1727 1735 10.1097/01.RVI.000018362.17771.B0 16371542

[B25] Boss A Clasen S Kuczyk M Schick F Pereira PL Image-guided radiofrequency ablation of renal cell carcinoma Eur Radiol 2007 12 725 733 10.1007/s00330-006-0415-y 17021704

[B26] Chang IA Considerations for thermal injury analysis for RF ablation devices Open Biomed Eng J 2010 12 3 12 20300227 10.2174/1874120701004020003 PMC2840607

[B27] Liu Z Ahmed M Sabir A Humphries S Goldberg SN Computer modeling of the effect of perfusion on heating patterns in radiofrequency tumor ablation Int J Hyperther 2007 12 49 58 10.1080/02656730601094415 17575723

[B28] Lobo SM Liu ZJ Yu NC Humphries S Ahmed M Cosman ER Lenkinski RE Goldberg W Goldberg SN RF tumour ablation: computer simulation and mathematical modelling of the effects of electrical and thermal conductivity Int J Hyperther 2005 12 199 213 10.1080/02656730400001108 16019848

[B29] Suarez AG Hornero F Berjano EJ Mathematical modeling of epicardial RF ablation of atrial tissue with overlying epicardial fat Open Biomed Eng J 2010 12 47 55 20300229 10.2174/1874120701004020047 PMC2841367

[B30] Elwassif MM Kong Q Vazquez M Bikson M Bio-heat transfer model of deep brain stimulation-induced temperature changes J Neural Eng 2006 12 306 315 10.1088/1741-2560/3/4/008 17124335

[B31] Consiglieri L Continuum models for the cooling effect of blood flow on thermal ablation techniques Int J Thermophys 2012 12 864 884 10.1007/s10765-012-1194-0

[B32] Ahmed M Liu ZJ Afzal KS Weeks D Lobo SM Kruskal JB Lenkinski RE Goldberg SN Radiofrequency ablation: effect of surrounding tissue composition on coagulation necrosis in a canine tumor model Radiology 2004 12 761 767 10.1148/radiol.2303021801 14990840

[B33] Tungjitkusolmun S Woo EJ Cao H Tsai JZ Vorperian VR Webster JG Thermal-electrical finite element modelling for radio frequency cardiac ablation: effects of changes in myocardial properties Med Biol Eng Comput 2000 12 562 568 10.1007/BF02345754 11094815

[B34] Ekstrand V Wiksell H Schultz I Sandstedt B Rotstein S Eriksson A Influence of electrical and thermal properties on RF ablation of breast cancer: is the tumour preferentially heated? Biomed Eng Online 2005 12 41 10.1186/1475-925X-4-41 16008834 PMC1188061

[B35] dos Santos I Haemmerich D da Silva Pinheiro C da Rocha AF Effect of variable heat transfer coefficient on tissue temperature next to a large vessel during radiofrequency tumor ablation Biomed Eng Online 2008 12 21 10.1186/1475-925X-7-21 18620566 PMC2500024

[B36] Ai HM Wu SC Gao HJ Zhao L Yang CL Zeng Y Temperature distribution analysis of tissue water vaporization during microwave ablation: Experiments and simulations Int J Hyperther 2012 12 674 685 10.3109/02656736.2012.710769 22946504

[B37] Yang D Converse MC Mahvi DM Webster JG Expanding the bioheat equation to include tissue internal water evaporation during heating IEEE Trans Biomed Eng 2007 12 1382 1388 17694858 10.1109/TBME.2007.890740

[B38] Yang D Converse MC Mahvi DM Webster JG Measurement and analysis of tissue temperature during microwave liver ablation IEEE Trans Biomed Eng 2007 12 150 155 17260866 10.1109/TBME.2006.884647

[B39] Pätz T Kröger T Preusser T Dössel O, Schlegel W Simulation of radiofrequency ablation including water evaporation, September 7–12, 2009, Munich, Germany. Volume 25/4 World Congress on Medical Physics and Biomedical Engineering 2010 Berlin Heidelberg: Springer 1287 1290 *IFMBE Proceedings*

[B40] Patz T Preusser T Composite finite elements for a phase change model Siam J Sci Comput 2012 12 B672 B691 10.1137/110853935

[B41] Cai ZH Song MY Zhang AL Sun JQ Xu LXM Numerical simulation of a new probe for the alternate cooling and heating of a subcutaneous mouse tumor model Numer Heat Tr a-Appl 2013 12 534 548 10.1080/10407782.2013.742809

[B42] Tungjitkusolmun S Staelin ST Haemmerich D Tsai JZ Cao H Webster JG Lee FT Mahvi DM Vorperian VR Three-dimensional finite-element analyses for radio-frequency hepatic tumor ablation IEEE T Bio-Med Eng 2002 12 3 9 10.1109/10.972834 11797653

[B43] Pennes HH Analysis of tissue and arterial blood temperatures in the resting human forearm (Reprinted from Journal of Applied Physiology, vol 1, pg 93–122, 1948) J Appl Physiol 1998 12 5 34 18887578 10.1152/jappl.1948.1.2.93

[B44] Chen X Saidel GM Mathematical modeling of thermal ablation in tissue surrounding a large vessel J Biomech Eng 2009 12 011001 19045917 10.1115/1.2965374

[B45] Hand JW Ledda JL Evans TS Temperature distribution in tissues subjected to local hyperthermia by RF induction heating Brit J Cancer 1982 12 31 35 PMC2149310 6950769

[B46] Gabriel S Lau RW Gabriel C The dielectric properties of biological tissues: II. Measurements in the frequency range 10 Hz to 20 GHz Phys Med Biol 1996 12 2251 2269 10.1088/0031-9155/41/11/002 8938025

[B47] Bhattacharya A Mahajan RL Temperature dependence of thermal conductivity of biological tissues Physiol Meas 2003 12 769 783 10.1088/0967-3334/24/3/312 14509313

[B48] Pop M Molckovsky A Chin L Kolios MC Jewett MAS Sherar M Changes in dielectric properties at 460 kHz of kidney and fat during heating: importance for radio-frequency thermal therapy Phys Med Biol 2003 12 2509 2525 10.1088/0031-9155/48/15/317 12953912

[B49] Mills R Self-diffusion in normal and heavy water in the range 1–45.deg J Phys Chem 1973 12 685 688 10.1021/j100624a025

[B50] Krynicki K Green CD Sawyer DW Pressure and temperature dependence of self-diffusion in water Faraday Discuss Chem Soc 1978 12 199 208

[B51] Nielsen LE The thermal and electrical conductivity of two-phase systems Ind Eng Chem Fundam 1974 12 17 20 10.1021/i160049a004

[B52] Jacques SL Prahl SA Modeling optical and thermal distributions in tissue during laser irradiation Lasers Surg Med 1987 12 494 503 10.1002/lsm.1900060604 3573921

[B53] Keangin P Rattanadecho P Wessapan T An analysis of heat transfer in liver tissue during microwave ablation using single and double slot antenna Int Commun Heat Mass 2011 12 757 766 10.1016/j.icheatmasstransfer.2011.03.027

[B54] Miklavčič D Pavšelj N Hart FX Akay M Electric properties of tissues Wiley Encyclopedia of Biomedical Engineering 2006 New York: Wiley 3578 3589

[B55] Iida H Aihara T Ikuta S Yamanaka N Effectiveness of impedance monitoring during radiofrequency ablation for predicting popping World J Gastroentero 2012 12 5870 5878 10.3748/wjg.v18.i41.5870 PMC3491593 23139602

[B56] Tateishi R Shiina S Teratani T Obi S Sato S Koike Y Fujishima T Yoshida H Kawabe T Omata M Percutaneous radiofrequency ablation for hepatocellular carcinoma - An analysis of 1000 cases Cancer 2005 12 1201 1209 10.1002/cncr.20892 15690326

[B57] Miao Y Ni YC Bosmans H Yu J Vaninbroukx J Dymarkowski S Zhang H Marchal G Radiofrequency ablation for eradication of renal tumor in a rabbit model by using a cooled-tip electrode technique Ann Surg Oncol 2001 12 651 657 10.1007/s10434-001-0651-y 11569780

[B58] Sun J Zhang A Xu LX Evaluation of alternate cooling and heating for tumor treatment Int J Heat Mass Tran 2008 12 5478 5485 10.1016/j.ijheatmasstransfer.2008.04.027

[B59] Chen C Zhang A Cai Z Sun J Xu LX Design of microprobe for accurate thermal treatment of tumor Cryo Letters 2011 12 275 286 21766157

